# A Semisupervised Learning Scheme with Self-Paced Learning for Classifying Breast Cancer Histopathological Images

**DOI:** 10.1155/2020/8826568

**Published:** 2020-12-08

**Authors:** Sarpong Kwadwo Asare, Fei You, Obed Tettey Nartey

**Affiliations:** ^1^School of Electronic Science and Engineering, University of Electronic Science and Technology of China, Chengdu, China; ^2^School of Computer Science and Engineering, University of Electronic Science and Technology of China, Chengdu, China

## Abstract

The unavailability of large amounts of well-labeled data poses a significant challenge in many medical imaging tasks. Even in the likelihood of having access to sufficient data, the process of accurately labeling the data is an arduous and time-consuming one, requiring expertise skills. Again, the issue of unbalanced data further compounds the abovementioned problems and presents a considerable challenge for many machine learning algorithms. In lieu of this, the ability to develop algorithms that can exploit large amounts of unlabeled data together with a small amount of labeled data, while demonstrating robustness to data imbalance, can offer promising prospects in building highly efficient classifiers. This work proposes a semisupervised learning method that integrates self-training and self-paced learning to generate and select pseudolabeled samples for classifying breast cancer histopathological images. A novel pseudolabel generation and selection algorithm is introduced in the learning scheme to generate and select highly confident pseudolabeled samples from both well-represented classes to less-represented classes. Such a learning approach improves the performance by jointly learning a model and optimizing the generation of pseudolabels on unlabeled-target data to augment the training data and retraining the model with the generated labels. A class balancing framework that normalizes the class-wise confidence scores is also proposed to prevent the model from ignoring samples from less represented classes (hard-to-learn samples), hence effectively handling the issue of data imbalance. Extensive experimental evaluation of the proposed method on the BreakHis dataset demonstrates the effectiveness of the proposed method.

## 1. Introduction

Breast cancer is one of the most frequent cancers among women and the second most common cancer globally, affecting about 2.1 million women yearly. Statistics from a global cancer report recorded that an estimated 627,000 women died from breast cancer in 2018 [1]. This figure is approximately 15% of all cancer deaths among women. Also, a recent report from the American Cancer Society's forecast for 2019 predicts that there will be almost 286,600 new cases of invasive breast cancer, about 63,930 new noninvasive cases, and about 41,760 deaths among women in the United States [2]. This worrisome trend necessitates the need for automated breast cancer detection and diagnosis [3]. Computer-aided detection or diagnosis (CAD) systems can contribute significantly in the early detection of breast cancer. Early detection is vital as it can help in reducing the morbidity rates among breast cancer patients [4].

Existing manual methods for breast cancer diagnosis include the use of radiology images in identifying areas of abnormalities. These images, however, cannot be used to accurately determine cancerous areas [5]. Biopsy [6] does help to identify a cancerous area in an image. Breast tissue biopsies help pathologists to histologically assess the microscopic structure and elements of breast tissues. The outcome of biopsy still requires a histopathologist to double-check on the results since a confirmation from a histopathologist is the only clinically accepted method. However, since the diagnosis provided by biopsy tissue and hematoxylin and eosin stained images is nontrivial, there is often some disagreements on the final diagnosis by histopathologists [7]. The drawbacks associated with the methods mentioned above drive the need for computer-aided systems for breast cancer diagnosis systems to improve diagnosis efficiency, increase the diagnosis concordance between specialists, reduce time, and lessen the burden on histopathologists [4, 8].

Deep convolutional neural networks (CNNs) have achieved tremendous successes in several disciplines including but not limited to object detection [9, 10], segmentation [11], and classification [12, 13]. Recent advancements in machine learning and deep learning in medical diagnosis are motivating lots of research in the classification of breast cancer histopathological images [14, 15]. The build nature of CNNs makes them capable of learning hierarchical feature representation from categorical data, and this is the underlying principle behind the success of CNNs in accomplishing tasks. In the specific case of breast cancer classification, existing work in the literature has adopted CNNs in achieving state-of-the-art results. Some of these methods mentioned in the literature are based on hand-engineered features [16–18]. However, methods that rely on hand-crafted features are inefficient and not robust, and they merely extract sufficient features that are beneficial in classifying histopathological images, not to mention that the entire process is a laborious and computationally expensive one. Other methods mentioned in the literature adopt deep learning approaches for classifying breast cancer histopathological images. Deep learning methods offer a better alternative to methods that rely on hand-engineered features, achieving excellent performances in many classification tasks [19–22]. Convolutional neural networks in particular have achieved state-of-the-art performances in classifying breast cancer histopathological images. In [23], the authors compared two machine learning schemes for binary and multiclass classification of breast cancer histology images. In the first approach, the authors extracted a set of hand-crafted features via bag of words and locality-constrained linear coding. They trained these features with support vector machines. Next, they experimented with a combination of hand-engineered features with a CNN as well as CNN features with the classifier's configuration. On the BreakHis dataset, the authors reported accuracy between 96.15% and 98.33% for binary classification and accuracy between 83.31% and 88.23% for multiclassification. Similar successes have also been reported in [8, 24, 25].

In spite of these successes, it is also pertinent to note that the deep layers associated with CNN models imply the fact that they require large amounts of well-labeled data during training to achieve satisfactory results. Training on relatively small amount of data leaves the models prone to overfitting and, subsequently, poor generalization. In the medical imaging domain, obtaining abundant labels for image samples is a major challenge, not to mention that a large amount of image samples are also required to aid in a model's ability to generalize well on data. Again, the process of labeling image samples is a time-consuming and an expensive one, requiring expertise knowledge. Existing methods mentioned in the literature that perform classification of histopathological images resort to training CNN models with random initialization and data augmentation techniques in a bid to improve a model's performance [23, 25, 26]. Such an approach enables a model to adapt to new data patterns on its own with augmented data samples that improve the number of training samples. These methods typically use only labeled data, since the learning process involved is a supervised one. However, an effective way of reducing labeling cost and generating more training samples is to make use of labeled and unlabeled data, via semisupervised learning (SSL) [27, 28]. Semisupervised learning aims to incorporate both labeled and unlabeled data in building better learners by fully considering the supervised knowledge delivered by labeled data and unsupervised data structure under unlabeled ones [27]. At the heart of semisupervised learning is training a learner on labeled data and using the learner to predict labels for unlabeled data. Moreover, compared to the process of obtaining well-labeled data, unlabeled data is rather inexpensive and abundant. Semisupervised learning algorithms have been adopted in some works mentioned in the literature for some classification tasks [27, 29–34].

In [35], the authors reported a cost-effective active learning approach for classifying deep images. Their proposed approach first progressively feeds samples from the unlabeled data into the CNN. Then clearly classified samples and the most informative samples are selected via a selected criterion and applied on the classifier of the CNN. The CNN model is then updated after adding user-annotated minority uncertain samples to the labeled set and pseudolabeling the majority certain samples. However, this approach acquires the least certain unlabeled examples for labeling and while simultaneously assigning predicted pseudolabels to most certain examples, and such a technique is not always helpful [36]. In [30], the authors use both labeled and unlabeled data for training a deep model across learning cycles. The authors employed both unsupervised feature learning and semisupervised learning. Unsupervised feature learning is used on all data once at the beginning of the active learning pipeline and the resulting parameters are used to initialize the model at each active learning cycle. The authors used semisupervised learning on all data at every learning cycle, replacing supervised learning on labeled examples alone, which is typical of tradition active learning methods. The approach adopted in this work parallels the works in [30, 37] in that a pseudolabel is generated for each unlabeled example but it differs from the work in [37] in that all unlabeled ones are pseudolabeled as opposed to only the majority high-confidence samples. This work employs semisupervised learning with self-training for training a classifier, rather than employing active learning. The work in [29] tackles the issue of classical multimedia annotation problems ignoring the correlations between different labels by combining label correlation mining and semisupervised feature selection into a single framework. Their approach utilizes both labeled and unlabeled data to select features while label correlations and feature corrections are simultaneously mined. In contrast, unlike selecting features via semisupervised learning, our work generates pseudolabels for the unlabeled samples and selects the most confident pseudolabeled samples via the pseudolabel generation and selection algorithm. By incorporating the self-paced learning concept into the selection process, the model learns samples from both well- and less-represented classes, which tackles the issue of model bias when selecting samples. The base model then learns features from both the labeled data and the selected pseudolabeled samples during training. We also solve the issue of class imbalance by introducing a class balancing framework. These two issues were not addressed in their work.

In [31], the authors proposed a semisupervised model named adaptive semisupervised feature selection for cross modal retrieval. In their semisupervised framework, the labels for unlabeled data are predicted by the graph-based label propagation. Then the unlabeled data with the predicted labels are combined with the labeled data to learn the mapping matrices. Meanwhile, the mapping matrices update the predicted label matrices, which can ensure that the raw feature distribution will be as consistent as possible with the semantic distribution in the subspace after several iterations. Our work parallels this proposed work with respect to predicting labels for unlabeled data and combining both the predicted labels with labeled data in updating training data for another iterative. The differences lie in the fact that our approach first uses the base learner to predict pseudolabels for the unlabeled samples after first training the learner with labeled samples, rather than graph-based label propagation. Then, a pseudolabel selection algorithm selects the most confident pseudolabeled sampled samples before updating the training samples with these selected pseudolabeled samples and labeled samples via self-training. This contrasts mapping matrices which are used to update the predicted label matrices in their approach. Again, our work focuses on generating confident pseudolabeled samples to augment the training data, making more reliable data available to the learner during training, as well as solving the issue of class imbalance in the data set while ensuring the fact that the model exhibits fairness in the selection process by learning from both well- and less-represented samples. Also, the work in [32] introduces a novel discriminative least squares regression (LSR) which equips each label with an adjustment vector. This technique avoids incorrect penalization on samples that are far from the boundary and at the same time facilitates multiclass classification by enlarging the geometrical distance of instances belonging to different classes. The authors assign a probabilistic vector fit each sample, hence ensuring the importance of labeled data while characterizing the contribution of unlabeled instance according to its uncertainty. Our approach primarily focuses on the generation of reliable pseudolabeled samples in augmenting the training data. The reliability of a pseudolabeled sample is determined by the pseudolabel selection algorithm which ensures the selection of pseudolabeled samples with the most confident probability. This prevents the situation where incorrectly labeled samples are added to the training samples. Also, our semisupervised learning approach hinges on the concept self-training and self-paced learning, which distinguishes our approach from the one reported in our work. The similarities lie in the fact that their proposed work and ours utilize both labeled and unlabeled data in the learning process.

To this end, this work proposes a novel semisupervised learning framework that uses self-training and self-paced learning (SPL) [38] to classify breast cancer histopathological images. Self-training is a semisupervised technique capable of learning a better decision boundary for labeled and unlabeled data. Self-training is accomplished by alternating between the generation of a set of pseudolabels corresponding to a large selection scores in the unlabeled-target domain and training a network (usually by fine-tuning) based on these selected pseudolabels and their corresponding pseudolabeled samples and labeled training data. The assumption here is that the target samples with higher prediction probability are right and have better prediction accuracy. In the proposed method, the process of generating and selecting pseudolabels is achieved via a novel pseudolabel generation and selection algorithm that selects only pseudolabels with the highest probability. The selection process is based on SPL, where in the initial learning stage, “easy” samples are selected and then “hard-to-transfer” samples are gradually added in a meaningful manner, making the classifier more robust. In a nutshell, the main contributions of this work are as follows:

  We propose a novel semisupervised learning framework that utilizes self-training with self-paced learning in classifying breast cancer histopathological images by formulating the problem as a loss minimization scheme which can be solved using an end-to-end approach.
  We introduce a novel pseudolabel generation and selection algorithm for selecting pseudolabels with relatively high-confidence probabilities to augment the training samples for retraining the model. In retraining the model, the optimization process begins by selecting pseudolabeled samples with relatively higher confidence (“easy” samples) then gradually adds “hard” samples to the training data. This ensures the selection of pseudolabels with high precision and prevents mistake reinforcement.
  To tackle the issue of class imbalance associated with self-training methods when generating and selecting pseudolabels, we implement confidence scores that use class-wise normalization in generating and selecting pseudolabels with balanced distribution.
  We obtain significant accuracy performance on the BreakHis dataset compared to the state-of-the-art approaches.

## 2. Methods

We provide an overview of the formulation of the problem as a loss minimization scheme which can be solved using an end-to-end approach. The concepts of self-training and self-paced learning as applied to the proposed scheme are also presented.

### 2.1. Preliminaries

For a given number of sample classes, the classification task is defined as a standard softmax loss on the labeled source data as inputs *x*_*s*_, *y*_*s*_ and the target data *x*_*t*_, *y*_*t*_:

(1) $$\begin{array}{c} L_{c}{\left(\chi ,y:\theta_{c}\right)}_{W}=-\sum_{k}1\left[y=k\right]\log\;P_{k}. \end{array}$$

In equation (1), the aim is to produce a classifier *θ*_*c*_ that can correctly classify target samples at the time of testing, with minimal loss. Nonetheless, based on the assumption that there is usually a limited amount of labeled target data (potentially from only a small subset of the categories of interest), effective transfer of representations becomes limited. Consequently, a classifier abandons the less-represented class samples in the learning process, focusing only on well-represented class samples. This ultimately impedes the classifier's ability to learn robust representations. The two key issues of learning the classifier lie in an effective formulation of a score function and a robust formulation of the loss function. Again, the robustness of a learner depends on the formulation of the loss function to relieve the influence of noisy and confusing data [39]. Moreover, the works in [40, 41] proved that the optimization problem of SPL solved by the alternative optimization algorithm is equivalent to a robust loss minimization problem solved by a majorization-minimization algorithm. In view of this, the problem is formulated as minimizing the loss function:

(2) $$\begin{array}{c} \min L_{c}{\left(W\right)}_{W}=-\sum_{l=1}^{L}\overset{N}{\underset{n=1}{\sum }}Y_{l,n}^{L}\log\left(P_{n}\left(W,I_{l}\right)\right)-\sum_{t=1}^{T}\sum_{n=1}^{N}Y_{t,n}^{T}\log\left(P_{n}\left(W,I_{t}\right)\right). \end{array}$$

*I*_*l*_ denotes the image in the source domain indexed by *l*=1,2,3,…, *L*. *𝒴*_*l*,*n*_ represents the true labels for the *n*th image (*n* = 1,2,…, N) for *I*_*l*_. W denotes the network weights. *P*_*n*_(*w*, *I*_*l*_) is the softmax output containing the class probabilities. Similar definitions hold for *I*_*t*_, *𝒴*_*t*,*n*_ and *p*_*n*_(*w*, *I*_*t*_) during evaluation. This problem formulation is different from [35] where the number of samples is represented as union of self-labeled high-confidence samples and manually annotated samples by an active user. We further formulate to minimize the loss function in equation (3). In the case where some target labels are unavailable, these labels are assumed to be hidden and the model learns from approximate target labels $\hat{𝒴}$ for $\hat{𝒞}$ (number of samples). In equation (3), $\hat{𝒴}$ is termed as pseudolabels:

(3) $$\begin{array}{c} \min L_{c}{\left(W,\hat{Y}\right)}_{W,\hat{Y}}=-\overset{L}{\underset{l=1}{\sum }}\overset{N}{\underset{n=1}{\sum }}Y_{l,n}^{L}\log\left(P_{n}\left(W,I_{l}\right)\right)\\ -\overset{T}{\underset{t=1}{\sum }}\overset{N}{\underset{n=1}{\sum }}{\hat{Y}}_{t,n}^{T}\log\left(P_{n}\left(W,I_{t}\right)\right). \end{array}$$

### 2.2. Self-Training with Self-Paced Learning

**Figure 1:**
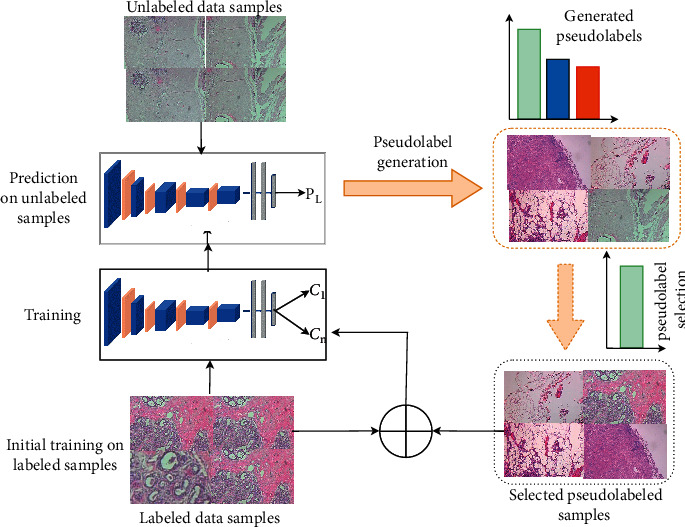
Workflow of the proposed approach. A deep CNN model is first trained with labeled data samples. The trained model is then evaluated on unlabeled data to generate pseudolabels for the unlabeled data. A pseudolabel selection algorithm that integrates a class balancing mechanism is used to select pseudosamples that have the highest confidence probability confidence score. The selected samples together with their pseudolabels are used to augment the training sample for the next training iteration and the cycle is repeated iteratively until a stopping criterion is met.

Semisupervised learning approaches typically adopt self-training to utilize unlabeled samples [42–45]. Based on the assumption of conventional self-training, an early mistake by the learner can reinforce wrong predictions into the training set for the next training iteration. To tackle this problem, a better alternative is to resort to adding samples by adopting an “easy-to-hard” approach via self-paced learning. The principal idea in self-paced learning is generating pseudolabels from “easy” predictions on the grounds that these approximate labels are right and correctly approximate the ground truth labels, then later exploring the “hard” or less-confident pseudolabels to update the model. The self-training process used in this work is outlined in Algorithm 1. A deep CNN model is first trained with labeled samples. The model then is then used to make predictions on the unlabeled data to generate pseudolabels *I*_*t*_. Similar to [30], all unlabeled samples are pseudolabeled. A novel selection algorithm with a class balancing mechanism is then used to select the nonannotated samples with the highest-confident probability predictions. These samples together with their approximated labels are added to the training set for the next training iteration. This cycle is executed iteratively until a stopping criterion is met. The overall workflow of our method is illustrated in Figure 1.

To incorporate the self-paced learning and self-training scheme, the loss function is modified as follows:

(4) $$\begin{array}{c} \min L_{c}{\left(W,\hat{Y}\right)}_{W,\hat{Y}}=-\overset{L}{\underset{l=1}{\sum }}\overset{N}{\underset{n=1}{\sum }}Y_{l,n}^{L}\log\left(P_{n}\left(W,I_{l}\right)\right)-\sum_{t=1}^{T}\overset{N}{\underset{n=1}{\sum }}\left[{\hat{Y}}_{t,n}^{T}\log\left(P_{n}\left(W,I_{t}\right)\right)+k_{c}Y_{t,n}^{\left(c\right)}\right]\\ \text{s.t.}\;Y_{t,n}\in \left\{e^{\left(i\right)}\in {\mathbb{R}}^{C}\right\},k_{c}>0. \end{array}$$

During training, *𝒴* is assigned to zero, implying that $\hat{𝒴}$ is ignored. To regulate the amount of pseudolabeled samples to be selected from the classes, *k*_*c*_ is introduced. The selection of a large quantity of pseudolabels is synonymous to a large value of *k*_*c*_. Adding *k*_*c*_ in equation (4) introduces a class-wise bias scheme that handles the issue of class imbalance when selecting pseudolabels. The pseudolabel selection process is accomplished in two steps: (1) initialize *W* and minimize the loss (in equation (4)) w.r.t. ${\hat{𝒴}}_{t,n}$ and (2) set ${\hat{𝒴}}_{t,n}$ and optimize the objective function in w.r.t. *W*. We considered the process of executing steps 1 and 2 as a single iteration and the two steps were repeated alternatively for several iterations. The task of solving Step 1 requires a nonlinear function and as such, Step 1 was reexpressed as

(5) $$\begin{array}{c} \min_{\hat{Y}}-\overset{T}{\underset{t=1}{\sum }}\overset{N}{\underset{n=1}{\sum }}\sum_{c=1}^{C}\left[{\hat{Y}}_{t,y}^{\left(c\right)}\log\left(p_{n}\left(C||w|,|I_{t}\right)\right)+k_{c}{\hat{Y}}_{t,n}^{\left(c\right)}\right]\\ \text{s.t.}\;{\hat{Y}}_{t,n}=\left[{\hat{Y}}_{t,n}^{\left(1\right)},\ldots ,{\hat{Y}}_{t,n}^{\left(c\right)}\right]\in \left\{e^{\left(i\right)}\in {\mathbb{R}}^{C}\right\},k_{c}>0. \end{array}$$

The introduction of a class-wise bias by normalizing class-wise confidence scores distinguishes this formulation from the one proposed in [21] where the authors adopted an *L*_1_ regularizer in a bid to avoid the scenario where most of the pseudolabels are ignored. In solving the pseudolabel framework optimizer, the work in [21] utilized the solver expressed in the following equation:

(6) $$\begin{array}{c} {\hat{Y}}_{t,y}^{\left(c*\right)}=\left\{\begin{array}{cc} 1, & \text{if }c=\arg\max\;p_{n}\left(c|w,I_{t}\right),p_{n}\left(c|w,I_{t}\right)>\;\exp\left(-k\right)\\ 0, & \text{otherwise}. \end{array}\right. \end{array}$$

With such a formulation, the process of generating and selecting pseudolabels hinges on the output probability (*p*_*n*_(*c|w*, *I*_*t*_)). Inherently, such an approach does not handle the issue of class imbalance. To resolve this, equation (3) is reexpressed as follows:

(7) $$\begin{array}{c} \min L_{c}{\left(W,\hat{Y}\right)}_{W,\hat{Y}}=-\sum_{l=1}^{L}\overset{N}{\underset{n=1}{\sum }}Y_{l,n}^{L}\log\left(P_{n}\left(W,I_{l}\right)\right)-\overset{T}{\underset{u=1}{\sum }}\sum_{n=1}^{N}\sum_{c=1}^{C}\left[{\hat{Y}}_{t,n}^{T}\log\left(P_{n}\left(W,I_{t}\right)\right)+k_{c}{\hat{Y}}_{t,n}^{\left(c\right)}\right]\\ \text{s.t.}{\hat{Y}}_{t,n}=\left[{\hat{Y}}_{t,n}^{\left(1\right)},\ldots ,{\hat{Y}}_{t,n}^{\left(c\right)}\right]\in \left\{e^{\left(i\right)}\in {\mathbb{R}}^{C}\right\},k_{c}>0. \end{array}$$

Minimizing the optimization framework in equation (7) was accomplished by using the loss function in equation (5) but with a solver that incorporates the class-wise normalizing term (different from the one proposed in [21]) expressed as

(8) $$\begin{array}{c} {\hat{Y}}_{u,y}^{\left(c*\right)}=\left\{\begin{array}{cc} 1, & \text{if }c=\arg\max\frac{p_{n}\left(c|w,I_{u}\right)}{\exp\left(-k_{c}\right)},\frac{p_{n}\left(c|w,I_{u}\right)}{\exp\left(-k_{c}\right)}>1\\ 0, & \text{otherwise}. \end{array}\right. \end{array}$$

The process of generating and selecting pseudolabeled samples is dependent on the normalized class-wise output (*p*_*n*_(*c|w*, *I*_*u*_))/(exp(−*k*_*c*_)) in equation (8). Using the normalized output ensures a balance towards classes with relatively low score but with a high intraclass confidence score during the process of assigning pseudolabels to an unlabeled sample.

To regulate the amount of pseudolabeled samples to be selected to update the model in each training iteration, *K*_*c*_ is set using the process in Algorithm 2. In finding and fixing a value for *K*_*c*_, the algorithm ranks the class C probabilities on all the image samples predicted as class C. *K*_*c*_ is set such that exp(−*K*_*c*_) is equivalent to the probability ranked at iteration (*p∗N*_*c*_), with *N*_*c*_ being the number of images predicted as class C. For each unlabeled sample, the maximum output probability *M* was taken in descending order and these probabilities are sorted out across all samples. Optimizing the pseudolabels resulted in the *p* × 100% most confident pseudolabeled samples to be used in training the model (where *p* is a scaled proportion between [0, 1]). Such a scheme ensures that the probability ranked at *p* × 100% is taken independently from each class to (1) threshold the confidence scores and (2) normalize the confidence scores. *p* is first initialized with 10% of the most confident predictions and at each additional round, the top 5% is added to the next pseudolabel generation and selection process.

## 3. Materials and Experiments

### 3.1. Dataset

**Figure 2:**
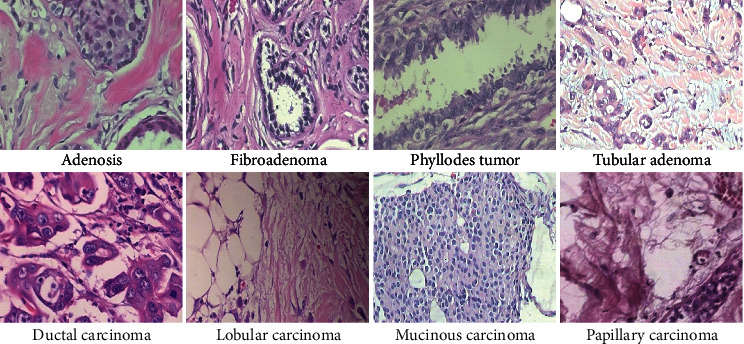
Sample image from each of the eight cancer subtypes in the BreakHis dataset. The images have subtle differences across classes due to their fine-grained nature, with different contrast and resolutions. These characteristics, coupled with the high coherency of the cancerous cells, make the dataset a challenging one. The images are obtained at a magnification factor of 200X.

We have carried out experiments on the BreakHis dataset [18]. The BreakHis dataset contains microscopic biopsy images of benign and malignant breast tumors totaling 7909 images. The image samples were generated from breast tissue biopsy slides, stained with hematoxylin and eosin (HE). Each image has a pixel size of 700 × 460 (in PNG format), with a 3-channel RGB, and 8-bit depth in each channel. The benign and malignant classes are each further subdivided into four distinct types. The subtypes for the benign class are adenosis, fibroadenoma, phyllodes tumors, and tabular adenoma. The malignant class subtypes are ductal carcinoma, lobular carcinoma, mucinous carcinoma, and papillary carcinoma. The images are obtained using four magnification factors −40X, 100X, 200X, and 400X. The images exhibit fine-grained characteristics with only subtle differences between images from different classes as well as high coherency, which is typical of cancerous cells. These factors, compounded with the fact that images in the same class have different contrasts and resolutions, make the BreakHis dataset challenging, not to mention the high imbalance in subtype classes (2,480 images belong to the benign class and 5,429 images belong to the malignant class).  Figure 2 shows sample images from each subtype class and Table 1 shows the distribution of images per each class.

### 3.2. Experimental Settings

The pretrained Inception_ResNetV2 [46], a variant of the Inception_V3 model [47], was used as the baseline model for all experiments. Inception_ResNetV2 is able to greatly improve classification and recognition performance at low computational costs. Input images are resized to 299 × 299 before being fed to the model. At the fully supervised learning phase, the baseline model is fine-tuned to initialize the model weights and also reduces variance. Fine-tuning of pretrained models has demonstrated to be an effective approach for achieving significantly higher results even on small-scale data. For the supervised learning phase, the model is trained for a total of fifty (50) epochs using the Adam optimizer [48], *β*_1_=0.9, *β*_2_=0.99 and an initial learning rate of 0.001 which is decayed via a polynomial decay scheduling (expressed in equation (9)). A polynomial decay scheduling allows the learning rate to decay over a fixed number of epochs:

(9) $$\begin{array}{c} \alpha =\text{initLR}*{\left(1-\frac{\text{epoch}}{T_{\text{epochs}}}\right)}^{p}, \end{array}$$

initLR is the base learning rate, *T*_epochs_ is the total number of epochs, and *p* is the exponential power, which is set to 1. The model is trained with a batch size of 32. Random rotation with a range of 90° and horizontal flipping have been implemented as data augmentation techniques to help combat overfitting. For the self-training phase, the model is also retrained with hyperparameters for top *K*_*c*_ using 5%, 10%, and 20% of the pseudolabeled samples of the unlabeled data. 70% of the data is used as training data and 30% is added to the test samples to be used as the unlabeled data for the self-training scheme. The training data was further split into 70 : 30 percent ratio as training and validation data, respectively. The model is trained for a total of 5 iterations during the semisupervised phase. We experimented with 5, 8, and 10 iterations and realized that not only did the 8 and 10 iterations take too much time to train, they also did not contribute significantly to the accuracy of the model compared to training for 5 iterations. To efficiently optimize training time, we decided to train for 5 iterations as this resulted in excellent accuracy within a limited time. Each experiment is repeated three times and the results are averaged. The iterations were stopped when there was no further improvement in accuracy.

The proposed approach does not add extra computational overhead during training, allowing training to be completed in an efficient manner. The averaged total training time for all experiments is shown in Tables 2 and 3, respectively. All experiments are carried out using Keras (version 2.2.4) with TensorFlow backend (version 1.12) and CUDA 9.0. Two RTX 2080 graphic cards, each with 8 GB memory and a 32 GB RAM, served as the hardware platforms. The evaluation metrics used in accessing the model were classification accuracy, precision, recall, F1-score, and confusion matrix. These parameters are related to the true positive (TP), true negative (TN), false positive (FP), and false negative (FN) rates, respectively. True positive measures how correctly a classifier predicts the positive class. True negative measures how correctly a classifier predicts the negative class. False positive measures how, incorrectly, a classifier predicts the positive class. False negative measures how, incorrectly, a classifier predicts the negative class.

## 4. Results and Discussion

The proposed scheme was evaluated using the top 5%, 10%, and 20% pseudolabeled samples. For purposes of reporting and investigation, we also report on values obtained when all pseudolabeled samples (100%) were used. We present and discuss results for both binary and multiclass classification tasks.

### 4.1. Binary Classification

The experimental outcomes for the binary classification task are shown in Table 4. For images with magnification factor of 40X, the best accuracy result was 99.52% when the top-10% pseudolabeled samples were selected. Similarly, for a magnification factor of 100X, the best accuracy result was 99.44% with the top-5% pseudolabeled samples. Using the top-10% pseudolabeled samples resulted in 99.48% accuracy for images with a magnification factor of 200X, and using the top-10% yielded an accuracy result of 99.47% with images scanned at 400X.

The generation and selection of the top *K*_*c*_ pseudolabeled samples via the proposed schemed was a vital key in controlling and determining the amount of pseudolabeled samples to be selected in updating the model at the next iteration. The selection scheme, coupled with the self-paced learning and self-training approach ensured that classes with the least representations which would have otherwise been ignored, was still selected and added to the training samples. This proved to be an effective and efficient step in the learning process. Again, the results in Table 4 show that selecting the top *K*_*c*_ pseudolabels proved to be a more effective approach rather than using all the pseudolabeled samples. The accuracy results obtained with the proposed approach show significant accuracy gains.

**Figure 3:**
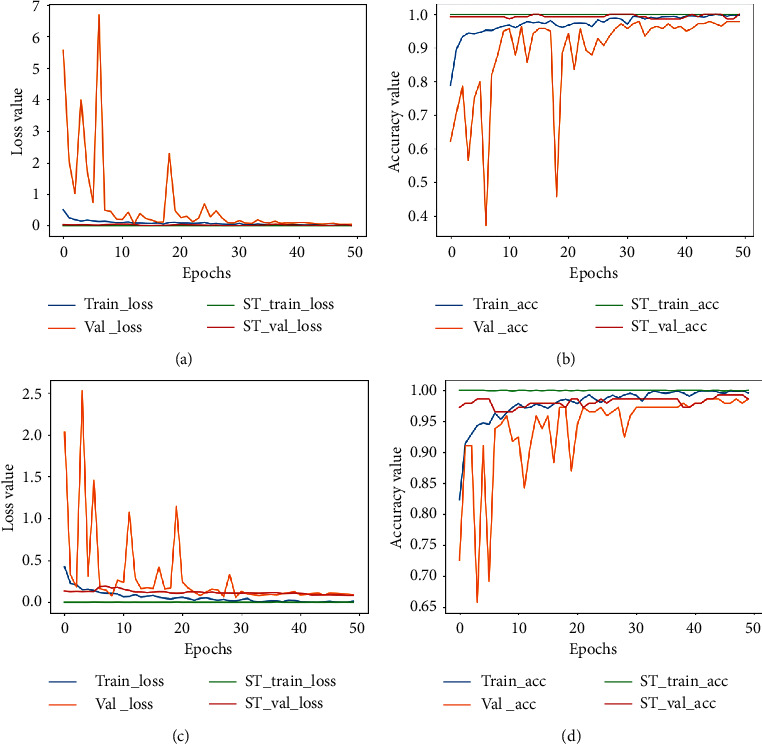
Accuracy plot for images scanned at 40X and 100X for the binary classification task. (a) The loss plot for 40X and (b) the corresponding accuracy plot. (c) The loss plot for 100X and (d) the corresponding accuracy plot. ST represents the self-training plot.

**Figure 4:**
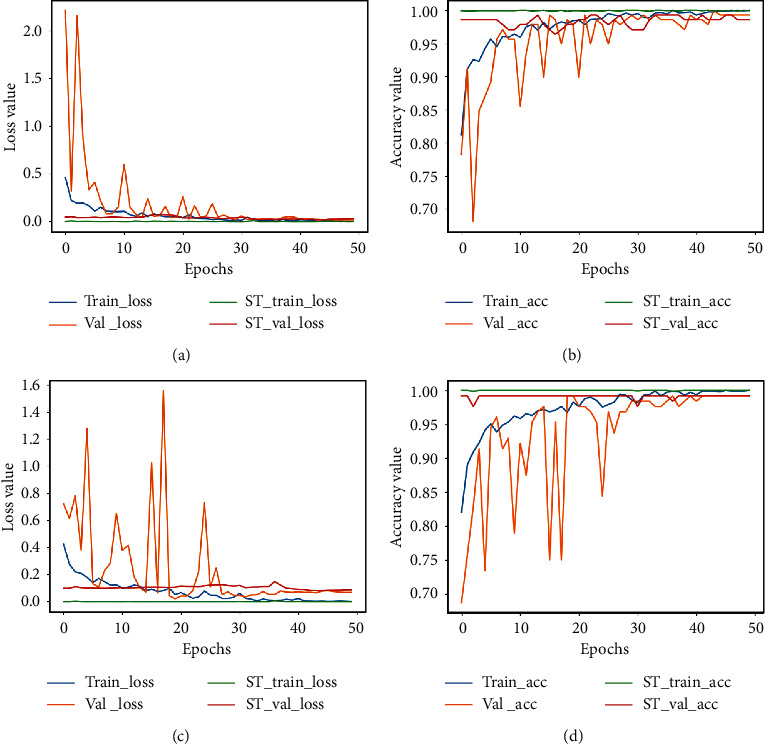
Accuracy plot for images scanned at 200X and 400X for the binary classification task. (a) The loss plot for 200X and (b) the corresponding accuracy plot. (c) The loss plot for 400X and (d) the corresponding accuracy plot. ST represents the self-training plot.

The accuracy and loss plots for 40X and 100X are shown in Figures 3 and 4 denotes plots for 200X and 400X, respectively. When training deep networks, overfitting remains a vital issue that needs to be addressed as it affects the ability of a trained model to generalize well on new data. It is observed from the plots that both accuracy and loss values were unstable until after epoch thirty (during the supervised learning stage). Values kept bouncing within different intervals from the start of training till the epoch thirty. We attribute this to the distance disparity between the source and target data. In fine-tuning a pretrained model on a secondary task, there is the assumption that the source and target domains are related to each other. However, in cases where this assumption is not met, brute-force transfer learning may not be successful and even in the worst case, degrading learning performance in the target domain [49].

**Figure 5:**
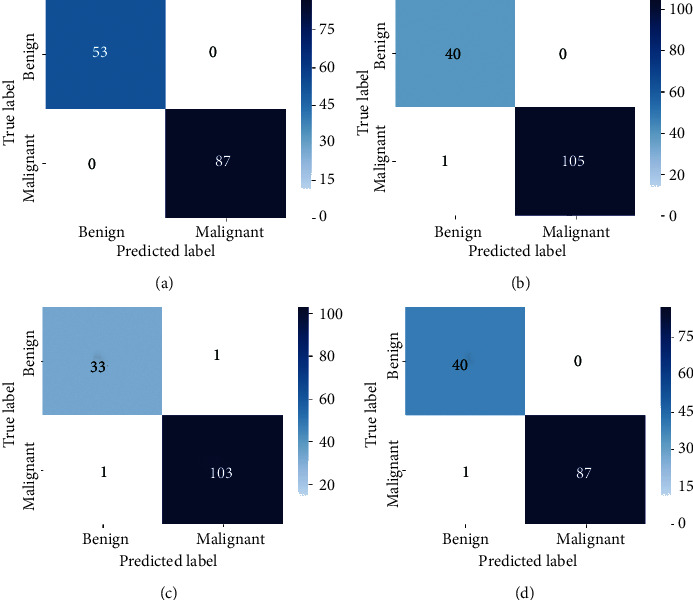
Confusion matrix for binary classification. (a) 40X. (b) 100X. (c) 200X. (d) 400X.

The pretrained model used as the baseline model was trained on the ImageNet dataset (which consists of natural images) as against the BreakHis dataset which contains breast cancer histopathological images. As such, at the start of supervised training stage, the model begins to learn the relatively new patterns from the target domain (breast cancer images) resulting in the spikes as depicted in the plots. However, past epoch thirty, a drastic drop in loss value is observed and the accuracy values increase steadily. At the end of epoch fifty, the loss value is greatly reduced and the training and validation accuracy (for both the supervised learning stage and the self-training stage) are almost aligned. This is an indication that the proposed approach also effectively curbs overfitting. The imbalanced nature of the BreakHis dataset implies that accuracy alone cannot be used to access the performance of the model. Results for precision, recall, and F1-score values are also presented in Table 5. The confusion matrices are also presented in Figure 5. The BreakHis dataset contains more samples for the malignant class compared to the benign class, and this is also reflected in the confusion matrices. Nonetheless, the selection process together with the class balancing framework adopted in this work ensured the fact that the model accurately classified the respective classes with minimal misrepresentations.

### 4.2. Multiclass Classification

The accuracy results for the multiclass classification are summarized in Table 6. For images scanned at 40X, the highest accuracy obtained was 94.28% when the top-10% pseudolabels were selected. For 100X, the best accuracy was 93.84% when the top-20% pseudolabels were selected. Selecting the top-5% pseudolabels yielded an accuracy of 94.93% for images scanned at a magnification factor of 200X. For images scanned at a magnification factor of 400X, the best accuracy was 93.75% when the top-10% pseudolabels were selected. Similar to the binary classification task, selecting the top *K*_*c*_ pseudolabels to augment the training samples in the next training iteration proved to be more effective than selecting all the pseudolabels. This outcome further rubber-stamps the significance of *K*_*c*_ in the proposed approach.

**Figure 6:**
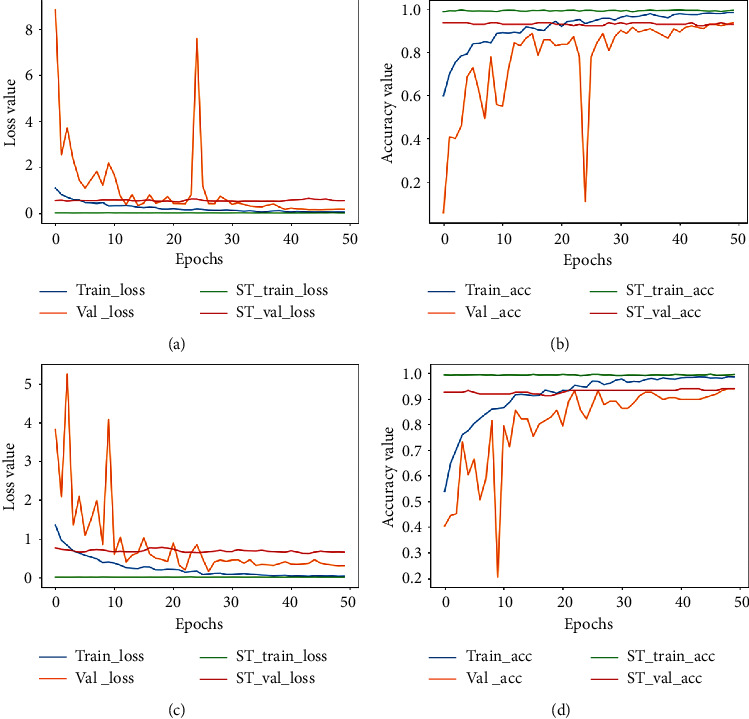
Accuracy and loss plot for images scanned at 40X and 100X for the multiclass classification. (a) The loss plot for 40X and (b) the corresponding accuracy plot. (c) The loss plot for 100X and (d) is the corresponding accuracy plot. ST represents the self-training plot.

**Figure 7:**
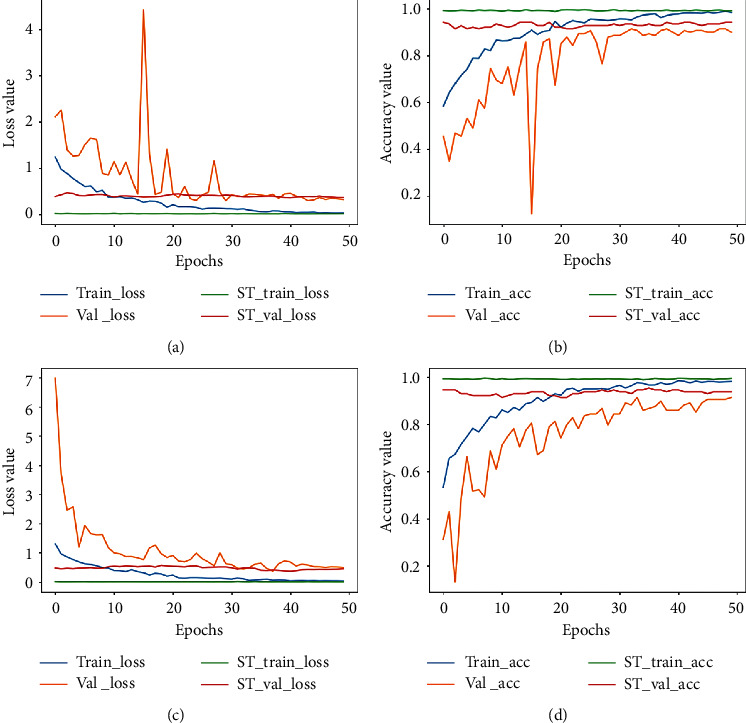
Accuracy plot for images scanned at 200X and 400X for the multiclass classification task. (a) The loss plot for 200X and (b) the corresponding accuracy plot. (c) The loss plot for 400X and (d) the corresponding accuracy plot. ST represents the self-training plot.

**Figure 8:**
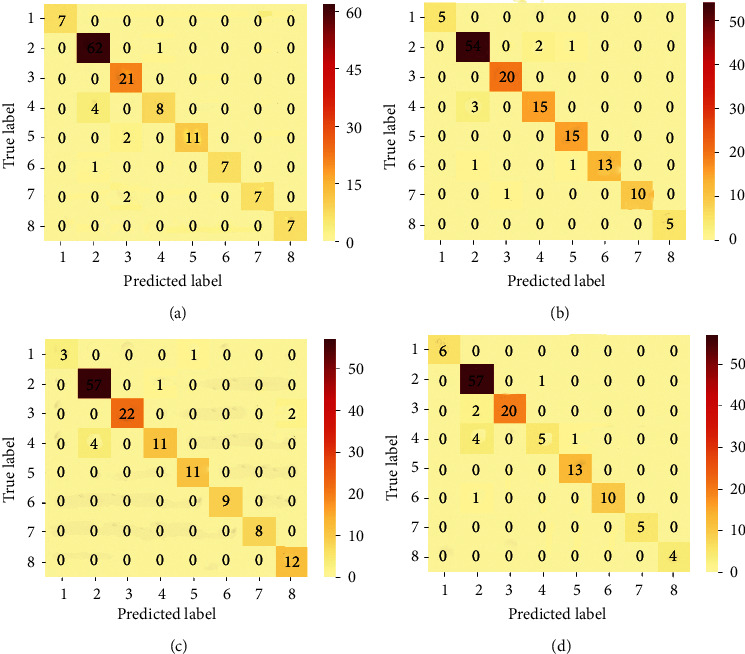
Confusion matrix for multiclass classification for the respective magnification factors. The imbalance in the sample distribution is evident in the plot. Nonetheless, there are not so many misrepresentations among classes. Order of class names: 1: adenosis, 2: ductal carcinoma, 3: fibroadenoma, 4: lobular carcinoma, 5: mucinous carcinoma, 6: papillary carcinoma, 7: phyllodes tumor, and 8: tubular adenoma. (a) 40X. (b) 100X. (c) 200X. (d) 400X.

The plots for loss and accuracy (for images scanned at 40X and 100X) are shown in Figure 6 and the corresponding plots for 200X and 400X are shown in Figure 7. The nature of the plots follow from the explanations provided for the binary classification plot. The precision, recall, and F1-score values are provided in Table 7 and the confusion matrices for all magnification factors are provided in Figure 8.

The confusion matrices also bring out the imbalance in the dataset. The ductal carcinoma class has more samples than the remaining classes with the adenosis class having the least number of samples. As a result, these two classes represent the most and least number of samples, as depicted in Figure 8. Again, the subtle nature of the appearance of the different images per different classes also does pose challenges for models in accurately discriminating between classes. In [23], the authors pointed out this difficulty, especially when discriminating between ductal carcinoma and lobular carcinoma as well as fibroadenoma and tabular adenoma. However, from the confusion matrices, it is observed that such misrepresentations are effectively handled by the proposed approach. Between ductal carcinoma and lobular carcinoma, an average of four samples are misrepresented while between fibroadenoma and tubular adenoma, only two samples are misrepresented for images scanned at a magnification factor of 200X.

The accuracy, precision, recall, and F1-score values as well as the confusion matrices all show the effectiveness of using *K*_*c*_ in determining the proportions of pseudolabels to be used in updating the model in each training iteration and also prove that adding samples in an “easy-to-hard” approach ensures that even the least-represented samples are still considered in the training process. Overall, these schemes resulted in the model being very versatile and robust even in the face of the similarities and coherence between the images samples in the dataset.

### 4.3. Comparison with Other Works

We compare the performance of the proposed approach with other works mentioned in the literature as shown in Table 8 for the binary classification task) and Table 9 (for the multiclass classification task), respectively. All these underlisted state-of-the-art methods were evaluated on the BreakHis dataset, offering a fair comparison and assessment with the proposed approach in this work. The work in [23] used a CNN model consisting of five convolutional layers and two fully connected layers for both binary and multiclass classification tasks. Using an ensemble method, the authors report accuracy of 98.33%, 97.12%, 97.85%, and 96.15% for magnification factors 40X, 100X, 200X, and 400X for the binary classification task. For the multiclass classification, they reported accuracy of 88.23%, 84.64%, 83.31%, and 83.39% for magnification factors of 40X, 100X, 200X, and 400X.

In [24], the authors proposed a structured deep learning model for classifying breast cancer histopathological images. In their work, the authors considered the feature space similarities of histopathological images by leveraging intra- and interclass labels as prior knowledge. They also adopted a data augmentation scheme that generated more data for the model during training. Using a pretrained deep CNN model as their base network, the authors reported accuracy of 95.8%, 96.9%, 96.7%, and 94.9% for the binary classification task. For the multiclass task, they reported accuracy of 92.8%, 93.9%, 93.7%, and 92.9% for magnification factors of 40X, 100X, 200X, and 400X, respectively. It can be observed that their approach yielded a 0.06% gain in accuracy for images scanned at 100X for the multiclass task compared to our approach. The data augmentation approach used in their work amassed more data for model during the fine-tuning stage compared to our approach and their overall approach was a supervised one (meaning only labeled data was used) as opposed the semisupervised fashion in ours (SSL dwells on the assumption that there are more unlabeled samples than labeled samples [27]). That notwithstanding, our approach yielded significant accuracy improvements for all the other magnification factors.

In [51], the authors proposed a novel L-Isomap-aided manifold learning and stacked sparse autoencoder framework for a robust BC classification using HIs. The authors reported accuracy of 96.8%, 98.1%, 98.2%, and 97.5% for images with magnification factors 40X, 100X, 200X, and 400X, respectively. In [50], the authors used a CNN model to extract local and frequency domain information from input images for classifying breast cancer images on the BreakHis dataset. They report accuracy of 94.40%, 95.93%, 97.19%, and 96.00% for the binary classification task. These algorithms mentioned in the literature only utilize supervised learning approaches.

In this work, we have used 70% of the data for training at the supervised learning stage and the remaining 30% was added to the test set which was used as unlabeled data for the self-training stage. The selection of the most confident pseudolabeled samples to augment the training sample has been proven effective in providing the model with reliable samples, and ultimately expanding the training set, thereby making more data available to the model (to satisfy the hunger of deep models for more data). The effectiveness of the proposed method is evident in the results obtained, which depict significant accuracy improvements compared to the abovementioned methods which are mostly supervised learning approach where only labeled data was used. The proposed algorithm has been tested on breast cancer histopathological images since it is in line with our research objective. Therefore, we are quick to add that, the significance of the proposed algorithm is not limited or specifically designed for breast cancer classification. Based on the results obtained, we are confident that this algorithm can be extended to other classification tasks in medical imaging or computer vision that seek to employ semisupervised learning techniques in solving various tasks.

## 5. Conclusion

Obtaining a significant amount of well-labeled data in the medical domain is a challenging task and more tedious is the task of accurately providing labels to data. In this work, we have proposed a semisupervised learning scheme that integrates self-paced learning paradigm and self-training for training a model on both labeled and unlabeled data. Self-paced learning plays a vital role in curbing the issue of mistake reinforcement, where wrongly generated pseudolabels are reinforced into the training sample. In the light of selecting pseudolabels with the most confident probabilities, we show a novel selection algorithm was proposed to present the CNN model with only the most confident pseudolabels. Experimental results obtained using the top 5%, 10%, and 20% generated pseudolabels for training showed significant accuracy improvements for both binary and multiclass classification task when compared with state-of-the-art approaches. For future work, we intend to incorporate diversity into the self-paced learning scheme and as well as incorporate the similarities in feature space of histopathological images. A combination of these elements into the self-paced learning scheme will result in a versatile and robust learner.

## Figures and Tables

**Algorithm 1:**
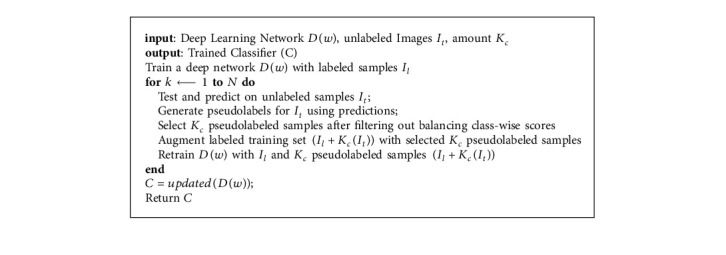
Self-paced learning workflow.

**Algorithm 2:**
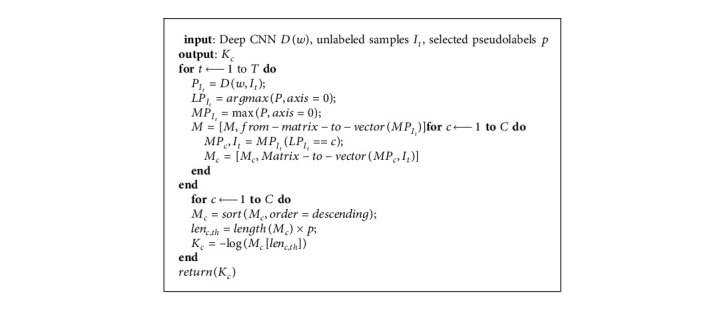
Determining *K*_*c*_.

**Table 1:** The distribution of images per individual subtype classes of the BreakHis histopathological images dataset.

Class	Subtype	Magnification factors			
		40X	100X	200X	400X
Benign	Adenosis	114	113	111	106
	Fibroadenoma	193	260	264	137
	Phyllodes tumors	149	150	140	130
	Tabular adenoma	109	121	108	115
Malignant	Ductal carcinoma	864	903	896	788
	Lobular carcinoma	156	170	163	137
	Mucinous carcinoma	205	222	196	169
	Papillary carcinoma	145	142	135	138

The distribution shows unequal number of image distribution per classes, resulting in class imbalance which makes the dataset a challenging one.

**Table 2:** Average training times for the binary classification task based on the amount of selected pseudolabels.

% of pseudolabels	40X	100X	200X	400X
K(top-5) pseudolabels	1 hour 59 min	2 hours 2 min	2 hours 4 min	1 hour 50 min
K(top-10) pseudolabels	1hour 56 min	2 hours 2 min	1 hour 59 min	1 hour 50 min
K(top-20) pseudolabels	1 hour 59 min	2 hours 3 min	2 hours	1 hour 53 min
All pseudolabels	1 hour 58 min	2 hours 4 min	1 hour 57 min	1 hour 49 min

min represents minutes.

**Table 3:** Average training times for the multiclass classification task based on the amount of selected pseudolabels.

% of pseudolabels	40X	100X	200X	400X
K(top-5) pseudolabels	2 hours	2 hours 5 min	2 hours 1 min	1 hour 47 min
K(top-10) pseudolabels	1hour 58 min	2 hours 5 min	1 hour 57 min	1 hour 49 min
K(top-20) pseudolabels	2 hours 1 min	2 hours	2 hours 2 min	1 hour 49 min
All pseudolabels	2 hours	2 hours 5 min	2 hours	1 hour 49 min

min represents minutes.

**Table 4:** Accuracy (%) performance for binary classification. Baseline indicates that the model was fine-tuned with labeled samples only. *K*_*c*_ (top-N) indicates the portion of the most confident pseudolabels used. Best results are indicated in italics.

ST approach	40X	100X	200X	400X
Baseline	97.14 ± 0.33	98.22 ± 0.40	98.55 ± 0.57	98.43 ± 0.44
*K*_*c*_(Top-5%) pseudolabels	99.28 ± 0.6	*99.44 ± 0.41*	99.03 ± 0.34	99.04 ± 0.73
*K*_*c*_(Top-10%) pseudolabels	*99.52 ± 0.33*	98.85 ± 0.32	*99.48 ± 0.30*	*99.47 ± 0.37*
*K*_*c*_(Top-20%) pseudolabels	99.27 ± 0.37	97.95 ± 0.01	98.79 ± 0.68	98.92 ± 0.22
All pseudolabels	98.09 ± 0.21	98.20 ± 0.13	98.5 ± 0.72	98.69 ± 0.58

**Table 5:** Precision (Prec.), recall (R), and F1-score (F1) values for binary classification.

Mag. factor	% of pseudolabels	Prec. (%)	R (%)	F1 (%)
40X	*K*_*c*_(top-5%)	99.50	99.23	99.38
	*K*_*c*_(top-10%)	99.89	99.79	99.81
	*K*_*c*_(top-20%)	99.50	99.21	99.36
	All pseudolabels	98.72	98.63	98.49

100X	*K*_*c*_(top-5%)	99.73	99.58	99.69
	*K*_*c*_(top-10%)	99.28	99.17	99.23
	*K*_*c*_(top-20%)	98.62	98.24	98.71
	All pseudolabels	99.12	99.06	99.19

200X	*K*_*c*_(top-5%)	99.43	98.91	99.18
	*K*_*c*_(top-10%)	99.84	99.80	99.49
	*K*_*c*_(top-20%)	99.27	99.00	99.13
	All pseudolabels	99.18	99.10	99.22

400X	*K*_*c*_(top-5%)	99.40	99.17	99.20
	*K*_*c*_(top-10%)	99.85	99.77	99.54
	*K*_*c*_(top-20%)	99.25	99.18	99.21
	All pseudolabels	99.20	99.00	99.14

**Table 6:** Accuracy (%) for multiclass classification.

ST approach	40X	100X	200X	400X
Baseline	91.42 ± 0.54	89.04 ± 0.70	90.07 ± 0.21	90.70 ± 0.63
*K*_*c*_(top-5%) pseudolabels	94.27 ± 0.28	91.78 ± 0.61	*94.93 ± 0.17*	92.97 ± 0.37
*K*_*c*_(top-10%) pseudolabels	*94.28 ± 0.29*	92.46 ± 0.48	94.32 ± 0.22	*93.75 ± 0.72*
*K*_*c*_(top-20%) pseudolabels	94.14 ± 0.14	*93.84 ± 0.35*	91.48 ± 0.28	92.19 ± 0.16
All pseudolabels	92.87 ± 0.71	90.41 ± 0.63	92.19 ± 0.38	91.40 ± 0.11

Baseline indicates that the model was fine-tuned with labeled samples only. *K*_*c*_ (top-N) indicates the portion of the most confident pseudolabels used. Best results are indicated in italics.

**Table 7:** Precision (Prec.), recall (R), and F1-score (F1) values for multiclass classification.

Mag. factor	% of Pseudolabels	Prec. (%)	R (%)	F1 (%)
40X	*K*_*c*_(top-5%)	94.96	94.71	94.67
	*K*_*c*_(top-10%)	95.15	94.78	94.80
	*K*_*c*_(top-20%)	94.63	94.55	94.59
	All pseudolabels	93.25	93.0	93.21
100X	*K*_*c*_(top-5%)	91.85	91.71	91.89
	*K*_*c*_(top-10%)	93.14	92.85	92.38
	*K*_*c*_(top-20%)	94.24	94.71	94.33
	All pseudolabels	90.63	90.39	90.51
200X	*K*_*c*_(top-5%)	95.85	95.90	95.56
	*K*_*c*_(top-10%)	95.47	94.91	95.32
	*K*_*c*_(top-20%)	91.44	91.07	91.64
	All pseudolabels	92.65	92.0	92.75
400X	*K*_*c*_(top-5%)	93.48	93.23	93.51
	*K*_*c*_(top-10%)	94.36	94.28	94.38
	*K*_*c*_(top-20%)	92.69	92.14	92.32
	All pseudolabels	90.63	90.57	90.41

**Table 8:** Accuracy comparison with some state-of-the-art algorithms for the binary classification task on the BreakHis dataset.

Ref.	Mag. fac.	Acc. (%)	Prec. (%)	R (%)	F1 (%)
Nahid and Kong [50]	40X	94.40	94.00	96.00	95.00
	100X	95.93	98.00	96.36	97.00
	200X	97.19	98.00	98.20	98.00
	400X	96.00	95.00	97.79	96.00
Han et al. [24]	40X	95.8 ± 3.1	—	—	—
	100X	96.9 ± 1.9	—	—	—
	200X	96.7 ± 2.0	—	—	—
	400X	94.9 ± 2.8	—	—	—
Pratiher and Chattoraj [51]	40X	96.8	—	—	—
	100X	98.1	—	—	—
	200X	98.2	—	—	—
	400X	97.5	—	—	—
Bardou et al. [23]	40X	98.33	97.80	97.57	97.68
	100X	97.12	95.58	96.98	97.77
	200X	97.85	95.61	99.28	97.41
	400X	96.15	97.54	96.49	97.07
*K*_*c*_(top-10% pseudolabels)	40X	99.52 ± 0.33	99.50	99.23	99.38
*K*_*c*_(top-5% pseudolabels)	100X	99.44 ± 0.41	99.73	99.58	99.69
*K*_*c*_(top-10% pseudolabels)	200X	99.48 ± 0.30	99.84	99.80	99.49
*K*_*c*_(top-10% pseudolabels)	400X	99.47 ± 0.37	99.85	99.77	99.54

Acc. denotes the accuracy, Prec. is the precision, *R* is the recall, and F1 is the F1-score.

**Table 9:** Accuracy comparison with some state-of-the-art algorithms for the multiclass classification task on the BreakHis dataset.

Ref.	Mag. fac.	Acc. (%)	Prec. (%)	R (%)	F1 (%)
Han et al. [24]	40X	92.8 ± 2.1	—	—	92.9
	100X	93.9 ± 1.9	—	—	88.9
	200X	93.7 ± 2.2	—	—	88.7
	400X	92.9 ± 1.8	—	—	85.9
Bardou et al. [23]	40X	88.23	84.27	83.79	83.74
	100X	84.64	84.29	84.48	84.31
	200X	83.31	81.85	80.83	80.48
	400X	83.98	80.84	81.03	80.63
*K*_*c*_(top-10% pseudolabels)	40X	94.28 ± 0.29	95.15	94.78	94.80
*K*_*c*_(top-20% pseudolabels)	100X	93.84 ± 0.41	94.24	94.71	94.33
*K*_*c*_(top-5% pseudolabels)	200X	94.93 ± 0.17	95.85	95.90	95.56
*K*_*c*_(top-10% pseudolabels)	400X	93.75 ± 0.72	94.36	92.28	94.38

Acc. denotes the accuracy, Prec. is the precision, R is the recall, and F1 is the F1-score.

## Data Availability

The data used in this work are available from [18] (DOI: https://doi.org/10.1109/TBME.2015.2496264).
